# Motivations for Use, User Experience and Quality of Reproductive Health Mobile Applications in a Pre-Menopausal User Base: A Scoping Review

**DOI:** 10.3390/healthcare13080877

**Published:** 2025-04-11

**Authors:** Alissa Kazakoff, Marissa L. Doroshuk, Heather Ganshorn, Patricia K. Doyle-Baker

**Affiliations:** 1 Human Performance Lab, Faculty of Kinesiology, University of Calgary, Calgary, AB T2N 1N4, Canada; alissa.kazakoff@ucalgary.ca (A.K.); marissadoroshuk@ucalgary.ca (M.L.D.); 2 Libraries and Cultural Resources, University of Calgary, Calgary, AB T2N 1N4, Canada; heather.ganshorn@ucalgary.ca; 3 Alberta Children’s Hospital Research Institute, University of Calgary, Calgary, AB T2N 1N4, Canada; 4 School of Planning, Architecture, and Landscape, University of Calgary, Calgary, AB T2N 1N4, Canada

**Keywords:** menstrual cycle, menstrual cycle tracking applications, fertility applications, reproductive health applications, mHealth, mobile applications

## Abstract

**Background:** The global mHealth market is growing at an unprecedented rate and is expected to reach an estimated value of $187.7 billion by 2033, with many apps now addressing women’s health and the menstrual cycle. This scoping review (ScR) aimed to comprehensively assess and describe the existing peer-reviewed literature on motivations for use, user experience, and reproductive health app quality. **Methods:** The protocol and review were conducted according to the JBI methodology and PRISMA guidelines for scoping reviews. Studies published in English since 2010 were included and searched in MEDLINE, Embase (Ovid platform), Scopus (Elsevier), ACM Digital Library, and IEEE Xplore. Studies were screened independently by two reviewers and the data explored through charting and synthesis. **Results:** Data were extracted from 58 papers published in English between 2014 and 2023. Several major themes related to motivations for app use, user experience, and app quality were identified and are reported on. **Conclusions:** Users were motivated to engage in reproductive health apps for education, contraception, and conception. This ScR identified several benefits, such as improving menstrual health literacy. We also identified limitations of current reproductive health apps that adversely affect user experience. Recommendations for future studies include increasing diversity, exploring perspectives of different user groups, and investigating the role healthcare providers may have in app development and patient education.

## 1. Introduction

The global mobile or mHealth market is growing at an unprecedented rate and is expected to reach an estimated value of $187.7 billion by 2033, according to Market.Us [1]. A large proportion of these developing technologies and apps are now addressing women’s reproductive health and the menstrual cycle with interest, use, and targeted funding increasing over the last five years [2,3,4]. Menstrual health is recognized as a vital sign of gynecological and overall health, and therefore several benefits of integrating mHealth into the daily lives of smartphone users have been identified [5]. Tracking health and menstrual cycle metrics can empower individuals by increasing awareness and understanding of their menstrual cycle. Many authors have also stated that tracking can provide preparation for different cycle phases, contraception, and fertility, improving health-related behaviors, and informing conversations with healthcare providers [4,6,7,8,9]. Despite these stated benefits, attrition is a key challenge for apps, with 71% of app users disengaging within 90 days of downloading the app [10,11].

To optimize the benefits of health tracking, it is important to understand factors of technology that motivate use and features that increase desirability for long-term use of an app [12]. Recent studies have outlined several factors that engage users in order to increase their likelihood of continuing app use. These include perceived personal and health benefits, app accuracy, usability, and user-friendly design [8,11]. Many menstrual cycle tracking apps, however, lack scientific validity or evaluation and do not have a standardized language to describe or characterize menstrual experiences [4,8,13]. These shortfalls and lack of inclusive designs can negatively influence the reliability of app predictions, while alienating users of gender and sexual minorities [6,14]. Therefore, this scoping review (ScR) aimed to comprehensively assess and describe the existing literature on motivations for use, user experience, and reproductive health app quality. The goal of this review was to provide insight into factors affecting app user experience and contribute to improved app design and development, thus meeting the technology needs of menstruating individuals [15,16].

## 2. Materials and Methods

The protocol and ScR were conducted according to the Joanna Briggs Institute (JBI) methodology for scoping reviews described in the 2020 JBI Manual for Evidence Synthesis [17] and reported according to the Preferred Reporting Items for Systematic Reviews and Meta-Analysis Protocols checklist (PRISMA–ScR) [18] (Table S1). The final protocol was registered and published (https://dx.doi.org/10.11575/PRISM/41536; accessed on 5 July 2023).

### 2.1. Information Sources and Search Strategy

#### 2.1.1. Preliminary Search

We followed the JBI guidelines/ScR beginning with a preliminary search of MEDLINE, and [5,6,8,9,14,15,16] articles were identified to ensure existing evidence was sufficient to perform a ScR. Several published ScRs were identified that were similar to our topic, such as that of Earle et al., 2021 [19] which provided an overview of the literature on mHealth apps with menstruation and fertility functions. However, to our knowledge, none were identified that explored both motivations for use and user experience of menstrual cycle tracking apps.

Text words contained in the titles and abstracts of relevant articles and index terms used to describe the articles were employed to develop a full search strategy for MEDLINE using keywords and controlled vocabulary terms related to the following concepts: menstruation/fertility and mobile apps. The search strategy, including all identified keywords and index terms, was adapted for the following specific databases so that overlap between databases would be kept to a minimum: Embase (Ovid platform), Scopus (Elsevier), ACM Digital Library, and IEEE Xplore. Complete search strategies for each database can be found in Appendix A. Only studies published in English were included to manage the volume of literature and ensure a manageable workload. Those studies published since 2010 were included as this timeline considered the introduction of smartphones and smartphone apps onto the market. Grey Literature was not searched as we were concerned that information about apps produced by some organizations may have specific agendas or interests which could lead to bias in the information. Therefore, we included only peer-reviewed research with a focus on traditional academic sources.

#### 2.1.2. Types of Source

This ScR included all published quantitative, qualitative, and mixed-method peer-reviewed studies. Any pre-prints or conference proceedings from the general medical literature were excluded. However, conference proceedings from computing literature were included, as that discipline often publishes full conference papers.

#### 2.1.3. Study/Source of Evidence Selection

**Figure 1:**
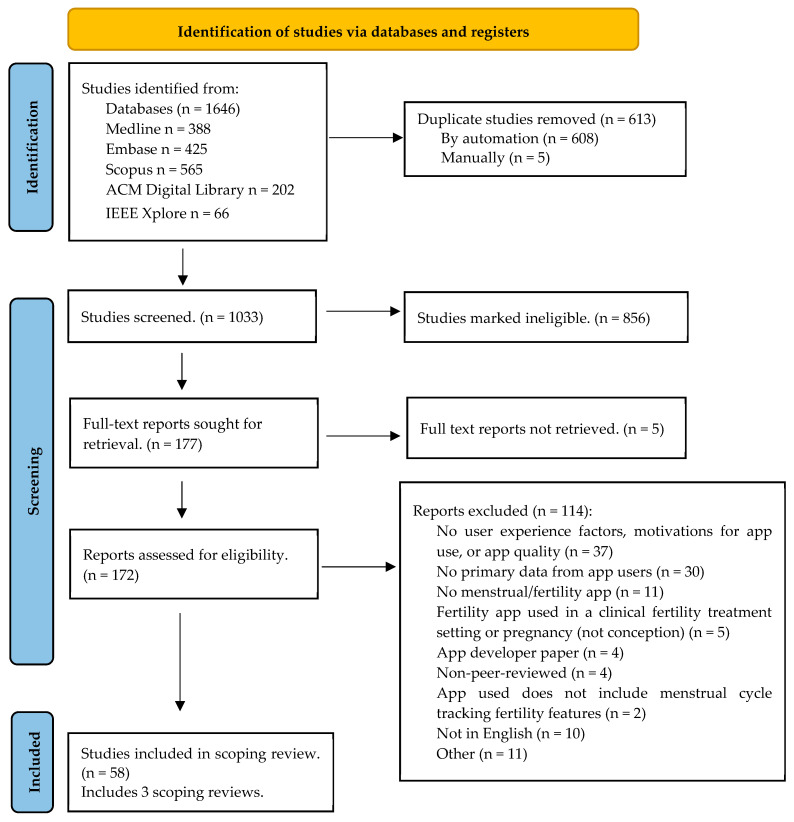
Screening studies, search results, study selection, and inclusion process [20]. For more information, visit: http://www.prisma-statement.org/ (accessed on 2 July 2024).

All identified citations were uploaded into Covidence (www.covidence.org; accessed on 4 June 2023) and duplicates were removed following the search. A pilot test of 50 titles/abstracts was completed by two independent reviewers (A.K., M.L.D.) against the ScR inclusion criteria with an 87% agreement. The remaining titles/abstracts and full texts of selected citations were assessed in detail against the inclusion criteria by the same reviewers. The results of the search, study inclusion process, and reasons for excluding sources of evidence in full text that did not meet the inclusion criteria are reported in Figure 1. Disagreements that arose between the reviewers at each stage of the selection process were resolved through discussion or with an additional reviewer (PKD-B).

#### 2.1.4. Inclusion and Exclusion Criteria

The area of focus for this ScR was defined within the following PCC (population, concept, context) framework [19].

Population: All studies with apps that serve a pre-menopausal menstruating population were included, regardless of geographic location. Population eligibility included menstruators and menstruating persons (i.e., people who menstruate but do not define themselves as women), with all gender identities eligible.

Concept: This scoping review investigated the existing literature on menstrual cycle tracking app use and factors contributing to user retention, such as motivations for the use of the app, user experience, and app quality. All studies investigating apps that serve a pre-menopausal menstrual cycle app user base were included, given that relevant data may be included even in studies where the ScR research question is not the focus.

Context: Motivations for use, user experience, app quality. The inclusion and exclusion criteria were established to reflect the PCC framework and included papers on menstrual cycle tracking apps used by a menstruating pre-menopausal female user base. The exclusion criteria included literature older than 2010, mHealth without menstrual cycle or fertility tracking options, and a menopausal population. The initial data extraction yielded 108 papers. We employed an iterative approach to data extraction and found that the inclusion and exclusion criteria were too broad. Many studies did not have primary data from app users, app assessments were completed by research teams, or apps were described from the developer’s perspective. The inclusion and exclusion criteria were subsequently revised to narrow the scope to include only papers that described direct user motivations for use and experience with menstrual cycle tracking apps. The final inclusion and exclusion criteria are outlined in Table 1.

#### 2.1.5. Charting the Data: Extraction and Presentation

One author (AK) extracted the data and organized it in an Excel spreadsheet with modifications as necessary, while extracting data from each included evidence source. Several categories were collapsed for clarity during the extraction process. The final extracted data focused on the three main outcomes: motivations for tracking, user experience, and app quality.

## 3. Results

After removing 613 duplicates (608 automatically and 5 manually), the initial search yielded 1033 studies for title and abstract screening. Of these, 856 were excluded (see Figure 1). This initially resulted in 172 articles in full-text screening. However, after narrowing the scope, data were extracted from 58 papers, of which three were scoping reviews.

All studies were in English and published between the years 2014 and 2023. Most studies (n = 35) were from Western/Commonwealth countries (USA, Canada, UK, Australia, New Zealand). Other locations of participants included Europe (n = 11), Korea and Japan (n = 5), Jordan (n = 3), India (n = 3), Brazil (n = 2), and countries in Africa (n = 1). Several study designs were identified and included quantitative (n = 27) (e.g., cross-sectional surveys), qualitative (n = 17) (e.g., semi-structured interviews), mixed methods (n = 11), and scoping/literature reviews (n = 3). Details on each study’s aim and apps included are presented in Table 2.

PRISMA 2020 flow diagram for new systematic reviews, which includes searches of databases.

### 3.1. Reproductive Health Apps

This ScR included studies on users of reproductive health apps. These applications perform a wide range of menstrual and fertility health functions [7,58]. Some common features include inputting symptoms and recording menstrual events or fertility indicators, such as basal body temperatures [11]. Apps may market themselves as fertility or menstrual apps but allow users to shift between contraception or conception goals [28]. In addition to menstrual cycle tracking, apps that support fertility awareness-based methods (FABM) are commonly used [3,19,64]. Ford et al. [33] defined reproductive health apps as “applications that have to do with the female reproductive system and may include features like menstruation tracking/calendar, pregnancy, or contraception/birth control”. When planning this ScR, we initially described the apps in question as “menstrual cycle tracking apps” to capture functions related to female reproductive health. While completing data extraction, no standardized term for reproductive health apps was used, and we identified many different synonymous terms in this field of research (Table 3). Thus, we employed the Ford et al. [33] definition, as it encompasses the diverse functions of these apps.

Fifty percent of the studies (29/58) described apps as menstrual cycle tracking apps and 36% (21/58) focused on fertility apps. Seven studies (12%) acknowledged the functions of menstrual tracking and fertility goals, highlighting the interconnectedness of the different elements of female reproductive health. Two studies were the exception, with the first reporting on male and female college student’s interest in a sexual health application which included menstrual tracking functions [57], and the second study instructing their participants to sign up for Clue, a commercially available menstrual tracking app used as a scientific research tool [46]. In summary we present a comprehensive overview of the current state of app tracking from the user perspective by including all studies describing apps with diverse functions related to female reproductive health.

#### Reproductive Health App Recruitment and Demographics

In general, these reproductive health app studies included high participant numbers, especially when recruiting via apps with large user databases [70,71]. For example, Alvergne et al. [21] analyzed data from 865 users recruited through Clue, Ponzo et al. [56] recruited 1867 participants through the Flo app, Bradley et al. [26] collected data on 45,360 users of the Ovia Fertility app, and Berglund Scherwitzl et al. [24] sent surveys to 4054 users registered for the Natural Cycles app. Social media recruitment methods resulted in even larger participant pools, such as Symul et al. [65], with 200,000 observations for the Sympto and Kindara apps. Epstein et al. [6] included 690 survey responses in their study, recruited via Facebook, Twitter, and Redditt. Haile et al. [40] 2018 recruited 356,520 participants for the CycleBeads app via a Facebook campaign across African countries (Egypt, Ghana, Kenya, Nigeria, and Rwanda), India, and Jordan. The Pregnancy Study Online (PRESTO) reported 10,599 completed surveys using a Facebook recruitment campaign [40,63]. A proportion of 20 or 34% of the studies recruited participants through social media, and 11 or 19% recruited through apps or app databases, but many included both recruitment techniques. Despite these large recruitment numbers, several studies reported dropout and loss to follow-up rates ranging from 38 to 70% [24,46,60].

Demographic information of participants was included in most studies and the majority of studies recruited only female participants or people who self-identify as period-tracking app users [22]. Five studies, however, investigated couples’ fertility app use or mHealth app features in addition to menstrual cycle tracking [19,33,46,57,64]. Only four studies reported gender identities outside the male/female binary [7,35,44,52]. In studies that enrolled both sexes, women comprised up to 73% of participants [23,57]. The mean age of the participants in the studies was within the age range of 24–31 years old unless the study specifically recruited college students or teenagers [3,9,15,21,24,25,27,29,37,46,48,57,60]. Seventeen (29%) studies reported including participants over 40 years old. Symul et al. [65] described the typical fertility app user as a 30-year-old with a “healthy” BMI living in a European or North American country. Similarly, other authors noted that their participant pools were primarily represented by the “W.E.I.R.D.” (Western, Educated, Industrialized, Rich, Democratic) demographic [6,21,72]. Studies that did not include the W.E.I.R.D. terms identified similar attributes of their participants. For example, Levy and Romo-Aviles [7] noted that the academic, white, middle-class network of the researchers influenced their participant demographic. This demographic makeup was common—many participants were described as white, educated, middle-class, partnered heterosexual females from European or commonwealth nations [15,33,36,37,41,44,48,57,63,64]. One study that recruited from a bleeding disorder clinic saw a more diverse user base, with only 34% of their participants identifying as Caucasian [30]. The study by Haile et al. [40] specifically recruited participants from seven non-Western countries: Egypt, Ghana, India, Jordan, Kenya, Nigeria, and Rwanda.

### 3.2. Themes

Several themes related to the ScR objectives (motivations for app use, user experience, and app quality) were identified during the analysis process. Although the themes aligned with the original protocol, we became aware of common threads that were not considered when conceptualizing the review. These included subthemes, such as the limitations of user experience and details relating to app quality. The remaining results are organized according to these themes (i.e., 1. motivations for app use, 2. user experience, and 3. app quality), along with the additional sub-themes.

#### 3.2.1. Motivations for App Use

A 2021 ScR by Earle et al. [19] described several reasons for reproductive health app use that center around understanding and observing menstrual cycle app phases, contraception, conception, and supporting fertility treatment or communication with healthcare providers, and explained that these motivations can change and evolve over time. Our ScR contains overlapping studies with Earle et al. but also includes other studies which identify additional reasons, such as assessing cycle normalcy and irregular patterns, record-keeping, self-diagnosis, mood tracking and premenstrual syndrome (PMS) symptoms, source of information for fertility care, birth control reminders, preparing for periods, self-knowledge and body exploration [7,9,33,44,49,54,58,60,62]. Tuli et al. [66] described menstrual tracking as akin to practicing body autonomy, especially for users nearing menopause and prioritizing pregnancy prevention. One study that did not overlap, by Richman et al. [57], reported an interest in sexual health management via period tracking, birth control reminders, STI and pregnancy symptom checks. Haile et al. [40] reported that women in Ghana, Kenya, Nigeria, and Rwanda used the CycleBeads app for pregnancy prevention, but users in India, Jordan, and Egypt used the app for conception.

##### Apps as a Non-Hormonal Alternative

There is a growing interest in pregnancy prevention using fertility apps as a non-hormonal alternative, in part due to concerns about hormonal birth control and side effects [19,29,31,36,39,40,64]. For some users, fertility apps can serve as a tool for bodily self-awareness while transitioning from hormonal contraception [31]. Individuals looking to conceive have also expressed the desire to do so “naturally” with the support of apps or use these to aid in conception efforts during fertility treatments [11,25,36,39]. Currently, 25% of Ovia Fertility users report conception in the app [26]. Similarly, 13.3% of respondents in the da Cunha Pereira study reported using natural contraception, 60.5% of participants expressed an interest in it, and 35.5% of participants would like to use natural contraception [29]. However, only 23.8% of current fertility app users are very confident that the app can help them avoid pregnancy, and 43.6% of those intending to use an app in the future are unsure if it could help them avoid pregnancy [64]. Some users involved in fertility treatments used the apps as a means for information, support, and regaining a sense of control, especially when dissatisfied with the information offered at fertility clinics [44,51,64]. Jukic et al. [48] also reported that 61% of their respondents using fertility apps had a history of miscarriage, and Schantz et al. [11] reported that apps may over-select users who are sub-fertile or looking to facilitate conception.

#### 3.2.2. User Experience: Strengths

Two major factors were identified contributing to the positive experience of reproductive health app users: management and education. As previously mentioned, apps are increasingly viewed by their user base as viable contraceptive methods. Berglund Scherwitzl et al. [24] reported that up to 83% of women were happier with apps compared to their previous method of contraception, and 88% would recommend this method to a friend. Sharing well-liked tracking methods between women appears to be common and occurs through friends and family, but especially from mother to daughter [6,9,11,30,40,60,62].

##### Cycle Management

Apps serve as a convenient management and calendar tool that can be integrated into daily routines [22,30,37,44,68]. Users appreciate apps that allow diverse functions to organize and prepare for their cycle events, view past data, and send data to and communicate with healthcare professionals [23,25,35,58,59,68]. Users of the Flo app reported that features, such as period predictions, symptom tracking, fertile day predictions, and health assistance helped them feel supported in managing and being prepared for their body’s symptoms [56]. Andelsman [22] described this as a “conversion of inner processes into actionable evidence”. This kind of support leads to users feeling more open about their symptoms, understanding their bodies, and feeling emotionally prepared for their periods [15,35,49,56]. The users of Flo in the Ponzo et al. [56] study were most positively impacted and less likely to take days off work for cycle-related issues. Fertility app users also had a favourable view of apps at the start of the fertility process, when they were more likely to feel a sense of control, or when their goals were achieved [28,32].

##### Educational Tool

Another outcome of using apps to manage both the menstrual cycle and symptoms is from the perspective of their use as an educational tool [7,33,37,56,61,73]. Currently a lack of menstrual literacy exists, especially in developing countries [74]. Despite this literacy gap, apps are reported as being used to access information easily [25,59,66,73]. Ford et al. [34] reported that app users improved their contraceptive knowledge by 21% compared to non-users. In Japan, Song and Kanaoka [61] argued that apps may be a tool for users to improve their lifestyle, alleviate symptoms of depression, and seek gynecological help after learning about PMS through the app. This is especially important for fertility apps and individuals or couples trying to conceive due to identified gaps in fertility knowledge, conception probabilities, and fertility treatments [25,33,62,67]. Several studies emphasize the educational benefits of fertility apps as a way to share public health messages and information on reproduction and improve the fertility knowledge of users [6,33,67]. For example, Sparidaens et al. [62] evaluated an online myFertiCare app and couples going through Assisted Reproductive Technologies expressed high information needs due to the stressful nature of the process, 79% reporting that the app increased their knowledge of fertility treatment. Other benefits include the cost-effectiveness of apps like Natural Cycles and accessibility to information in areas where traditional care might be inaccessible [34,36].

#### 3.2.3. User Experience: Limitations

Despite the benefits, many studies discussed the limitations relating to the current state of menstrual cycle and fertility tracking using apps. Some basic reasons for discontinuing app use include forgetting to enter data and technical difficulties with the apps [6,15,46,54,66]. Several studies identified that using reproductive health apps can invoke a negative emotional experience when the app’s predictions do not align with the true lived experience of the user, especially for users with irregular cycles [7,15,32]. Inaccurate predictions were described by Broad et al. [15], who reported that 54.9% of users get their periods earlier than predicted, and 73.1% get their periods later than predicted. As a result of inaccurate app predictions, users may develop distrust or experience frustration from the lack of individualized app predictions. This may lead to low expectations of the app and subsequently reduce user app input [6,11,15,37,44,54]. Misunderstanding app limitations [41,43] may also increase user distress concerning their reproductive health, and lead to seeking medical care as a result [7,15,58]. Apps with symptom-tracking features could also lead to exacerbated symptom reporting, health anxiety, and increased health-seeking behaviors, as stated by Mackrill et al. [53]. Josephy et al. [47] demonstrated that, when comparing smartphones to paper diaries, the median number of symptoms reported was significantly higher with the smartphone app group. Additional studies have described that extensive tracking could lead to feeling overwhelmed, defined as “tracker fatigue” [19,31,41,44,75].

Currently, as apps establish de facto what a ‘normal’ cycle is, questions arise regarding their suitability for irregular cycles and the inappropriate use of such terminology, highlighting the unmet needs of diverse user groups [37,44,54,58]. For people attempting to conceive, apps can exacerbate the emotional toll of fertility struggles [25,32]. Four different types of terms were coined by Figueiredo et al. [32] to demonstrate this negative engagement with fertility data: burdened, obsessive, trapped, and abandoning. Individuals will often start out as positive and then transition through these negative engagement types when they experience stress and anxiety following a period of unsuccessful tracking (burdened) [32]. Individuals can become consumed with tracking and incorrectly interpret symptoms as signs of pregnancy in the absence of pregnancy (obsessive) [32]. In trapped engagement, they start to feel dependent on tracking, even in the face of feeling guilt, despair, and abnormality due to unsuccessful attempts at conceiving [32]. The result is that individuals may eventually abandon tracking and app engagement, possibly as a defiant move [32,45].

### 3.3. App Quality and Features

Our review of app user experience revealed several key themes concerning app quality. These themes reflect the current shortcomings of reproductive health apps and outline the main features that app users seek in future app developments.

#### 3.3.1. Limited Regulation of Apps

Currently, no regulatory body oversees the quality of menstrual cycle and fertility apps entering the market, suggesting that the quality of apps—especially fertility tracking apps—is likely to be variable [19,37,50,54,59]. Evaluation studies reported that 95% of the 108 free menstrual tracking apps do not have professional involvement or cited literature [76], and only 3.8% of users indicated that the app they used had medical approval [54]. Although apps are not regulated as medical devices or meant for such use, only 10% of apps mention this [14,77]. Currently, Natural Cycles and Clue are the only apps approved by the FDA, and Natural Cycles is cleared in Europe as a contraceptive [14,27,37]. However, Karasneh et al. [8] stated that Natural Cycles was assessed by its founders, highlighting potential bias in some app evaluations. Evidence suggests that the contraceptive effectiveness of Natural Cycles is comparable to that of other methods [24,69]. However, maintaining a low failure rate using Natural Cycles necessitates a commitment from users to abstain from intercourse or use other contraception during fertile days, which may be difficult for some [3,24,69]. In another fertility app (CycleBeads) evaluation study, Shelus et al. [60] reported that 60% of users abstained or used condoms, while 46% employed the withdrawal method, and 12% used emergency contraception.

#### 3.3.2. Incorrect Predictions

To elaborate further, because apps lack rigorous evaluation, they are often incorrect in their predictions [8]. For example, the ScR by Earle et al. [19] reported that only 9% of apps accurately predict the fertile window. This finding is corroborated by several additional studies that investigated app accuracy and found that a majority do not perform adequately—approximately 18–20% are of good quality, provide accurate information, or predict ovulation and the fertile window correctly [25,33,34,43,64,76,78]. Many apps are not transparent about their limitations or how predictions are calculated and may end up misleading their users [7,43]. Apps often use scientific jargon for marketing, and this may misrepresent their true capabilities [19,64]. Moreover, there is a gap in users’ knowledge regarding app features and how to interpret predictions [43]. For example, some users will use Fertility Awareness Based Methods with apps that are not intended for those functions [64]. While some users may abandon app use due to being skeptical of predictions and aware of inaccuracies, others continue to use and trust their apps [6,15,22,37,68]. This may lead to harm for users, such as unintended pregnancies, anxiety around distressing notifications, an app detecting early miscarriage, or not knowing when to seek further medical advice [36,44,64].

#### 3.3.3. Design Limitations

Many studies identified design limitations and concerns concerning menstrual tracking and fertility apps. These concerns encompass privacy issues, invasive advertisements, non-inclusive designs, and a lack of customization features. Several diverse aspects of privacy came out in users’ feedback. In some studies, privacy concerns were minimal, with as high as 83% of participants unconcerned [15,39]. However, users valued data security and privacy, and some reported feeling uncomfortable with third parties having access to data and their data being used beyond the app or leading to potential data leaks [6,7,23,41,44]. Currently there are minimal regulations and laws surrounding privacy protections for health information or women’s health apps, meaning that marketers could access this data [16,35,54,79]. Consequently, some users report receiving unwanted advertisements that feel too invasive and intimate [37,58,79,80].

A recurring common thread was the lack of inclusive designs and ethnic and gender diversity representation in apps [6,7,28,29,31,37,48,51,64,66]. Apps have continued to reinforce this issue through stereotypical female-gendered graphics and heteronormative designs that assume users are cisgender and heterosexual [6,35,52]. Because many menstrual and fertility apps target white, middle-class, heterosexual demographics, this perpetuates stereotypes related to “Western” settings, such as framing PMS as a negative event [21,41,65]. Other constructs reinforced through apps include framing irregular cycles as deviant, ageist designs not allowing for menopause transition, gendered pronouns, exclusion of gender minorities, and stigmatizing and insensitive jokes [22,35,39,66,68]. These issues alienate diverse users and do not account for life transitions and evolving goals [11,35,66]. Along these lines, users reported that current apps lack customization functions [30,35]. Users desire app designs that can be customized to provide personalized experiences, which include diverse populations with different or evolving goals and reproductive health stages [6,7,8,15,16,28,32,37,66]. Apps are sometimes oversimplified and limited in their functions in supporting complex symptom reporting, or users outside of their reproductive years [18,19,49,60]. There is also a desire for more comprehensive tracking across all aspects of health, including exercise, sleep, mental and physical health, emotions, and sexual activity [6,7,28,37,52]. Yet, simplicity and user-friendly designs remain important factors, and too many options and complexity can also lead to abandoning apps [8,11,19,44].

## 4. Discussion

We identified 58 studies addressing the motivations for use, user experience, and quality of reproductive health apps among a pre-menopausal user base. The mHealth market, as stated in our introduction, is currently experiencing significant growth and attention [1,81]. Additionally, with the release of ChatGPT in 2022, artificial intelligence and tech have become salient topics in health research [82]. Some of the highlighted benefits of incorporating mHealth into healthcare include empowering users through the accessibility of information, tracking and managing personal health, remote healthcare access, and reducing healthcare demand and costs [1,83,84,85]. These factors become relevant and important in the context of female reproductive health apps, especially now that menstrual health is recognized as a vital sign of overall health and is a global health priority [5,86]. With an estimated growth of 13 to 62 million active users of the Flo app alone between 2017 and 2024, there also appears to be a clear and increasing demand for reproductive health apps among users [87]. Our ScR findings support these trends, with many of the studies demonstrating high participation numbers, ranging between 865 and 356,520, when recruiting from apps such as Clue, Flo, Ovia, Natural Cycles, Sympto, and Kindara, social media, or a combination of methods [6,21,24,26,40,56,63,65,88]. Many women are known for being interested in participating in research for altruistic reasons [42] while others are influenced by free access for a given period of time [76], both of which were likely contributing factors to the large recruitment numbers.

### 4.1. Motivations for App Use

The motivations for using reproductive health apps have been well described in the literature and related ScRs, like that of Earle et al. [19]. Overall, motivations to use apps center around three core reasons that may change and evolve through different life stages: understanding the body and menstrual cycle, “natural” contraception, and conception [6,7,8,11,15,16,19,25,28,29,31,32,36,37,39,40,64]. Interestingly, two studies highlighted that apps may over-select users who have experienced miscarriages or fertility problems [11,48]. Future research should further explore users’ attitudes towards hormonal contraception and the impact it has on motivations to use reproductive health apps, as well as investigate whether people experiencing subfertility are more likely to use reproductive health apps.

#### 4.1.1. User Experience: Management, Education, Empowerment

Our inquiry into the experiences of reproductive health app users revealed an in-depth overview of the current state of reproductive health tracking. We identified positive aspects of user experience that relate closely to the initial motivation to use apps. On the other hand, our findings indicate that the currently available apps have limitations that may contribute to negative aspects of user experience. This offers insight into future research and recommendations for app improvements in order to serve users better with, importantly, better predictions. Since this field is constantly evolving, there is still a large potential for improvement and growth in app quality, which would benefit the user and, by extension, reproductive health app developers by improving retention.

Several reported positive experiences stem from fulfilling users’ initial motivations for using the apps and include two major factors: menstrual management and education. Women appreciate integrating tracking into daily routines to be prepared for their cycle events and to gather data that can be used in communication with healthcare professionals [22,23,25,30,35,37,41,44,56,59,68]. Reproductive health apps may be a key to increasing accessibility to menstrual and fertility education and supporting users’ reproductive health literacy. The potential impact of this is considerable—the education and validation of experiences through app tracking leads to feelings of empowerment in users. Through tracking, users not only learn more about their bodies but also see their data and symptoms represented in the app, validating their lived experiences and contributing to a feeling of appreciation for their cycles [7,31,66]. Some participants report feeling embarrassed about menstruation when they are young; therefore, apps can be a way to challenge social norms and the stigma around menstruation [22,35,66].

Considering the often high-stakes journey of conception, Hamper [41] describes fertility tracking as a “hope technology” that empowers women in their reproductive choices and prepares them for pregnancy. With the educational potential of fertility apps, users feel empowered if their information needs are met, leading to higher fertility literacy and better coping and feelings of control concerning the stresses of treatment [51]. An additional unexpected outcome was partner involvement in app use for contraception or conception. Since this often involves two people, users were motivated to use fertility apps and involve their partners in the process [31,32]. In one study, up to 89% of women discussed app use for pregnancy prevention with their partners, even reporting that it improved partner communication [60].

#### 4.1.2. App Limitations

These apps’ current limitations often hinder the potential for positive outcomes. Studies report that there is no regulation of reproductive health apps entering the market, leading to variable quality, little health professional involvement or medical approval, and minimal protection around privacy [16,19,35,37,50,54,59,76,79]. Many of these apps lack transparency about their limitations and privacy policies and misrepresent their capabilities, leaving users unaware of the lack of data protection [7,19,43,64]. For example, a class action lawsuit was recently launched in Canada against the Flo app for sharing sensitive health data with third parties, like Facebook [89]. This is concerning, because many app users value data security and privacy and are worried about data leaks and invasive advertisements [6,7,23,37,41,44,58,79].

When users recognize inaccuracies, a problematic sequence of events often results in attrition and discontinued use of the app [6,11,15,22,32,37,43,54]. Users with low menstrual literacy seek apps for information and are very trusting in the app’s predictions. However, because there is a gap in knowledge regarding app features and limitations, misaligned predictions can lead to harm and negative emotional experiences, like health anxiety, self-blame, unintended pregnancies, or increased stress around conception [7,15,32,36,43,44,58,64,72]. Some researchers [3,15,43] are recommending that users be aware of app limitations and do not rely on them. App developers should ensure transparency regarding predictions and privacy policies, strengthen data protection, address the emotional and mental aspects of tracking, and allow users to report inaccuracies [6,15,34,68]. If more apps seek FDA (USA Food and Drug Administration) or other approvals, this could also potentially increase quality and users’ trust in the apps [14,27,29,37].

#### 4.1.3. Diversity Issues

Many studies highlighted a lack of diversity. We observed that the demographic composition of apps may be partly influenced by the app designs that tend to reinforce a female-gendered, heteronormative, “Western” stereotypes target, subsequently attracting similarly defined users [6,21,35,41,52,65]. Apps also lack inclusive designs for diverse gender identities, users with irregular cycles, and those in life stages outside of the reproductive years [6,7,22,25,28,31,35,37,48,51,64,68]. As a result, this alienates and stigmatizes users who do not fit into the “typical” fertility app demographic, potentially further contributing to a homogeneous sample [11,49,60]. This lack of diversity in users and app design leads to missing perspectives of underserved user groups [86]. We observed also that users desire app designs that can be customized to provide personalized experiences for diverse populations with different or evolving goals and reproductive health stages [6,7,8,15,16,28,31,32,37,61].

From a big-picture perspective, some studies argued that self-tracking through apps can reduce the female body to a disorderly object that needs to be measured, controlled, and be under surveillance, again perpetuating cultural “Western” ideals and reinforcing social constructs around menstruation [21,22,31,32,41,42,45,58,66,90]. This can create tension between lived experiences that are complex, evolving, and uncertain in nature and the perfection and discipline often imposed by apps [31,41,45]. Additionally, using these apps requires a data entry commitment which can create additional work for women during a process that is meant to simplify day-to-day menstrual and fertility management [31,39,41].

We recommend that future research should explore the perspectives and needs of groups outside of the “Western” construct [25]. For example, several studies pointed out that Indigenous Māori people do not stigmatize menstruation and treat it as sacred [42,44,58]. People with religious backgrounds may have different goals and uses for reproductive health apps [6,31]. App developers should consider improving accessibility to a much broader base, which currently is a missed opportunity. This could include vulnerable populations who lack health care access, as well as individuals with sensory and cognitive disabilities, both of which may drive higher usage for apps and could contribute to a positive shift in the narrative around tracking.

#### 4.1.4. Role of Healthcare Professionals

Finally, amid the findings on app quality and desired features, the role of doctors and healthcare providers emerged. Because there is no standardized quality regulation, some studies argue that healthcare professionals should be involved in app development and evaluation, positively contributing to app quality [16,37,50,51]. Users also report valuing apps that are supported by research and vetted by healthcare providers [35,51,64]. When it comes to the doctor’s office, there appears to be an interest in integrating apps into the doctor–patient relationship [6,9,36,59,67]. Apps observed in this ScR are increasingly being used as accessible and empowering tools for managing reproductive health, sometimes in cases where users have felt unheard by healthcare providers [7,29,36,40]. Due to the widespread use of apps, some studies propose that healthcare providers have a responsibility to their patients to acknowledge their limitations, play a role in evaluating them, and counsel patients in their use [8,29,37,43,49,51]. This is especially important since apps are not currently at a level to replace in-person care [7]. Several studies highlighted that healthcare providers currently have limited knowledge about apps or can be dismissive of them and, therefore, rarely discuss them with their patients [9,36,64,65]. Future studies should investigate the current knowledge, opinions, and practices of healthcare providers regarding reproductive health apps.

### 4.2. Strengths and Limitations

A strength of this ScR is the breadth of information extracted from the studies (n = 58), which provides an in-depth overview of motivations for use, user experience, and app quality. This same wide scope of the topic made it difficult to narrow down the inclusion and exclusion criteria for the ScR. The broad objectives reached across both quantitative and qualitative study designs, and therefore the extracting and standardizing of information, was difficult, resulting in some studies contributing more to the analysis than others. The data extraction process was conducted by a single author (AK) and reviewed by another (PKD-B), yet it is possible that some information may have been overlooked.

There was a lack of standardized terminology regarding reproductive health apps as previously mentioned. This made it challenging to assess the functions of the included apps and make meaningful comparisons between studies. A future study should conduct a comprehensive review of the published terminology surrounding reproductive health apps to provide clarity on this issue. Another general limitation is the potential for selection bias, as many studies recruited participants through the apps themselves, meaning that these participants were already active app users and may have a pre-existing interest in using apps [24,25]. Future research should explore non-users’ attitudes to reproductive health apps. Finally, a significant strength of the app-based studies is the large number of participants.

## 5. Conclusions

This ScR identified 58 peer-reviewed studies addressing and describing the motivations for use, user experience, and the quality of reproductive health apps. Our findings demonstrated the current relevance of this topic, with many of the included studies reporting high participant numbers of app users. We found users were motivated to use reproductive health apps for education, contraception, and conception. Our ScR identified several benefits for using reproductive health apps, such as improving menstrual health literacy and helping users prepare for and feel empowered with their cycles. Despite the benefits, we found a number of limitations of current reproductive health apps that adversely affect user experience, which include a lack of regulation, predictions that lacked accuracy, and minimal population diversity. Recommendations for future studies include increasing diversity by exploring the needs and attitudes towards apps of underserved groups, perspectives on hormonal contraceptives and their impact on app use, the role that physicians and healthcare providers can have in app development, and patient education. Although we did not include in our search whether an app meets Human–Computer Interaction standards or whether AI-driven features improve engagement, this is also an area that requires careful consideration going forward [91]. Importantly, developers/designers should be aware of opportunities to prioritize transparency regarding their app functions and limitations, as well as being receptive to learning from and adapting to underserved groups. Overall, our ScR provides an updated insight into factors affecting user experience and contributes to improved app design and development, thus better meeting the technology needs of menstruating individuals [15,16].

## Figures and Tables

**Table 1:** Inclusion/Exclusion Criteria.

Inclusion	Exclusion
Menstrual cycle and fertility tracking apps	App developer paper
Menstruating pre-menopausal females	Does not include primary data from app users
	Fertility app used in a clinical fertility treatment setting or pregnancy (not conception)
	Menopausal population or app
	No mention of user experience factors, motivations for app use, or app quality
	App used does not include menstrual cycle tracking/fertility features
	No menstrual/fertility app
	Non-peer-reviewed (i.e., conference proceedings other than computing conferences)
	Not in English
	Older than 2010
	Other

**Table 2:** Study characteristics by author, year, country, aims, and associated apps/technology (n = 58).

Author (Year)	Country	Study Aims	App/Tech
Al-Rshoud (2021) [3]	Jordan	Examined the efficacy of fertility tracking mobile apps as a natural method of contraception.	Unspecified Fertility Awareness Method (FAM) Apps/Fertility Tracking Mobile Applications
Alvergne (2018) [21]	US, UK, Canada, Australia	Explored whether STIs lead to side effects in the days preceding menstruation and assessed digital health research capacity.	Clue
Andelsman (2021) [22]	Denmark, Netherlands	Investigated how menstrual cycles materialized within the contextual use of app-assisted period tracking.	Clue, My Calendar, Monthly Cycles, Period Tracker, Flo, Natural Cycles, FitrWoman
Anderson (2016) [23]	Australia	Conducted a qualitative analysis of users’ experiences with mobile health apps, their perceived advantages, and proposed recommendations for app enhancements.	Menstrual cycle monitoring (4 types, unspecified)
Berglund Scherwitzl (2016) [24]	Sweden	Presented a mobile application (Natural Cycles), outlined its core features and functions, and evaluated its contraceptive effectiveness.	Natural Cycles
Berglund Scherwitzl (2017) [23]	Sweden	Calculated the application’s perfect-use efficacy to re-examine its typical-use efficacy and failure rate method.	Natural Cycles
Blair (2021) [25]	UK	Investigated women’s use of FTA, their understanding of conception, and the demand for and acceptability of a potential natural conception prediction app.	Ovia Fertility, Glow, Flo, Maya, Ava
Bradley (2021) [26]	USA	Assessed the relationship between cycle length and variability and the time to conception using a mobile app.	Ovia Fertility
Broad (2022) [15]	UK	Described women’s real-life experiences with period tracker apps, their perceptions of the app and its ovulation information, and how the app’s accuracy in predicting period start dates influenced their feelings and behaviors.	Unspecified Period Tracker Apps
Bull (2019) [27]	Sweden	Examined the link between prior contraceptive choices and the app’s effectiveness, as well as the population-level effect of Natural Cycles on unintended pregnancy rates.	Natural Cycles
Costa Figueiredo (2021) [28]	USA	Examined the design of consumer-facing fertility apps to understand how they assisted menstruating individuals in achieving diverse fertility-related goals.	A total of 16 apps from Apple and 15 from Google
da Cunha Pereira (2022) [29]	Brazil	Evaluated the knowledge and interest of two groups—women and OBGYN residents—regarding natural contraception and smartphone applications.	Unspecified fertility awareness method smartphone apps
Dietrich (2017) [30]	USA	A digital strategy was implemented to improve patient adherence to medications and appointments while delivering educational outreach to individuals with heavy menstrual bleeding and bleeding disorders.	iPeriod application
Dudouet (2021) [31]	UK	Aimed to investigate personal experiences with using an app for contraception.	Natural Cycles, Kindara, Glow, Clue and Flow.
Earle * (2021) [19]	UK	Examined existing knowledge of the use of menstruation and fertility tracking apps.	Menstruation and fertility app trackers
Epstein (2017) [6]	USA	Provided insights into why and how women track their menstrual cycles, highlighted design challenges and concerns in digital tools, and offered guidance while challenging assumptions in the design of personal informatics tools.	Selected the 12 most reviewed apps.
Figueiredo (2018) [32]	USA	Explored self-tracking and emotions related to fertility, highlighting its highly personalized nature and the complexity of this health concern with limited personal control.	Fertility tracking apps
Ford (2020) [33]	Australia	Identified differences in fertility knowledge associated with the use of female reproductive health apps.	Reproductive health apps
Ford (2022) [34]	Australia	Reviewed peer-reviewed literature on fertility-based reproductive health apps and examined the information the apps provide.	Fertility apps
Fox (2020) [35]	USA	Explored efforts to revisit and reimagine menstrual tracking technology, focusing on mobile apps designed to document and quantify menstrual cycle data.	Period tracking technology/mobile applications
French (2022) [36]	UK	Investigated how women and their partners navigate (pre)conception healthcare and the role of Natural Cycles fertility awareness technology in this process.	Natural Cycles
Gambier-Ross (2018) [37]	UK (Scotland)	Examined women’s use of and interactions with FTAs to inform the design and development of the next generation of these tools.	Fertility Tracking Apps (FTA) Clue was most popular (12%)
Gazibara (2020) [38]	Serbia	Investigated the prevalence of menstrual cycle tracking app use among high school girls and identified factors associated with their usage.	Menstrual cycle tracking apps
Goncalves (2021) [9]	Brazil	Assessed the frequency and experiences of app usage among Brazilian women.	The most used apps were Flo (31.1%), My Calendar (25.8%) and Clue (24.9%).
Grenfell (2021) [39]	UK	Investigated the role of Natural Cycles in users’ and their partners’ (pre-)conception practices and experiences.	Natural Cycles
Haile (2018) [40]	African countries, India, Jordan	Conducted market tests of the CycleBeads app across seven countries.	CycleBeads app
Hamper (2020) [41]	UK	Examined women’s use of fertility apps during attempts to conceive and investigated how technological practices influence the understanding of bodies as reproductive.	Fertility tracking applications/fertility apps
Hohmann-Marriott (2021) [42]	New Zealand	Investigated app users’ experiences and perceptions, emphasizing menstrual app data within the contexts of menstrual health and digital health.	Menstrual-cycle-tracking apps: 22 were using general predictive apps and 3 had apps applying Fertility Awareness Based Methods.
Hohmann-Marriott (2022) [43]	New Zealand	Examined how app users who are not trying to conceive interpreted and utilized information from menstrual apps.	General purpose menstrual apps. Clue is most popular. Flo, Period Tracker, iHealth, Period Diary, My Calendar, and Eve. FABM apps: Ovagraph, My NFP, and Kindara.
Hohmann-Marriott (2023) [44]	New Zealand	Aimed to understand the role of menstrual tracking apps in addressing menstrual disorders and diseases in New Zealand by consulting expert stakeholders, including healthcare providers, app users, and patients.	Flo, Clue, Period Tracker, My Calendar, Period Diary, Balance, Apple Health, Fitbit, Lily, Ava, Daysy, Kindara
Homewood (2020) [45]	Denmark	Examined how data continues to influence individuals after they stop self-tracking and explored the ways this information manifests within lived experiences.	Clue
Jacobson (2018) [46]	USA	Compared patient satisfaction and compliance between mobile app reporting and paper reporting among menstruating adolescent girls.	PBAC diary in mobile app format
Josephy (2022) [47]	USA	Assessed the completeness and timeliness of data gathered through apps, text messages, and paper diaries.	Clue
Jukic (2023) [48]	USA	Analyzed the characteristics of users of a menstrual cycle tracking app among participants in an online research study.	Ovia Fertility
Karasneh * (2020) [8]	Jordan	Conducted a systematic review to identify period tracking smartphone apps and evaluate their composition, quality, and effectiveness.	49 available English language and free-to-download apps were included on period tracking
Ko (2023) [16]	Korea	Reviewed existing menstrual apps, focusing on their primary content and quality as evaluated by healthcare providers and consumers.	34 apps identified with the keywords “period” and “menstrual cycle” in English and Korean
Lee (2019) [49]	Korea	Investigated whether menstrual health mobile apps can be chosen based on users’ needs to influence health-related factors.	2 unspecified apps
Lee (2022) [50]	Korea, USA	Focused on refining and enhancing MASUN 1.0 while evaluating the feasibility and usability of the updated version, MASUN 2.0.	5 menstrual apps
Lemoine (2021) [51]	Canada	Identified the information needs of individuals seeking fertility services, analyzed the relationships between meeting these needs, psychological outcomes, and demographics, and discussed the implications for readability standards.	Infotility (informational web-based app)
Levy (2018) [52]	Austria, Spain	Explored users’ experiences with and reactions to gendered design in app-supported menstrual tracking.	Unspecified menstrual apps
Levy (2019) [7]	Austria, Spain, Italy	Investigated how period-tracking apps contribute to user empowerment and enhance menstrual health literacy.	App-supported menstrual tracking
MacKrill (2020) [53]	New Zealand	Examined the impact of a menstrual-monitoring app with a symptom tracker on symptom reporting.	Flo, Next Period
Nair (2023) [54]	India	Explored young women’s knowledge, attitudes, and practices concerning the use of mobile applications for menstrual tracking.	42.5% used some kind of health-tracking mobile app. Users of the MTA Flo had the highest representation (30.7%). The sample also had users of the Indian MTAMaya (17.9%)
Ozcelik (2020) [55]	Turkey	Compared effectiveness, satisfaction, and continuation rates between the traditional CycleBeads method and its smartphone application counterpart.	CycleBeads App
Ponzo (2022) [56]	UK	Assessed the impact of menstrual cycle-related disturbances on work productivity among Flo app users.	Flo Health App
Richman (2014) [57]	USA	Evaluated college students’ (1) sexual health behaviors, (2) mobile technology usage, and (3) interest in a mobile health app for improving and managing sexual health.	Not specified
Riley (2022) [58]	New Zealand	Aimed to expand the MTA study both empirically and theoretically by analyzing the experiences of five users managing PMS with MTA.	Clue, Flo
Robertson (2021) [59]	UK	Reviewed and summarized all digital support tools developed to date for fertility patients.	46 digital support tools for fertility patients
Schantz * (2021) [11]	USA	Reviewed and synthesized literature on MCTAs regarding their applicability in epidemiologic research.	Menstrual Cycle Tracking Apps (MCTA)
Shelus (2017) [60]	Kenya	Evaluated whether the app attracted new family planning users, examined user experiences, and analyzed how these experiences differed based on the channel through which women discovered the app.	CycleBeads app
Song (2018) [61]	Japan	Examined the effectiveness of a mobile application in mitigating mental and physical disorders and reducing labor productivity loss caused by menstruation-related symptoms among working women in Japan.	Karada-no-kimochi
Sparidaens (2023) [62]	Netherlands	Assessed the implementation of the myFertiCare app and its impact on couples’ knowledge of fertility treatment, perceived treatment burden, and experience of patient-centered care.	Online myFertiCare app
Stanford (2020) [63]	Canada, USA	Evaluated the use of mobile computing apps to estimate their impact on fecundability within an internet-based volunteer group of couples attempting to conceive.	Clue, Fertility Friend, Flo, Glow, Kindara, My Days, Ovia, Period Tracker
Starling (2018) [64]	USA	Identified women using or intending to use fertility apps for pregnancy prevention and examined their preferences and perceptions regarding app usage.	Period Tracker, Fertility Calendar, Fertility Friend, Natural Cycles, Ovia, Ovuline, Glow, Dot, Pink Pad Pro, OvaGraph, Kindara, iCycleBeads, Conceivable, Clue, 2DayMethod, and unnamed others
Symul (2019) [65]	150 countries (mostly Europe and Americas)	Characterized users and their tracking behaviors, providing an overview of the observations logged in the apps, and developed a statistical framework for estimating ovulation time based on self-reported data.	Sympto and Kindara, Methods
Tuli (2022) [66]	India	(1) Investigated how individuals at various stages of their menstrual journeys engage in tracking and the factors influencing their choices. (2) Examined how tracking practices evolve during the transition from menarche to menopause, reflecting different life decisions. (3) Explored experiences with and aspirations for digital menstrual trackers.	Digital menstrual trackers
Yokomizo (2021) [67]	Japan	Examined the potential of high-quality fertility treatment information to improve fertility treatment literacy within a large Japanese population.	Luna
Zampino (2019) [68]	Italy	Investigated how individuals engage with self-tracking technologies, reshaping the interaction between expert and lay knowledge.	Self-tracking technologies, apps to manage menstrual periods

Note: Created using the evidence table from the Schantz et al. (2021) [11] scoping review as a template. * scoping reviews. Abbreviations: Fertility Tracking Apps (FTAs), Menstrual Cycle Tracking Apps (MTAs/MCTAs). The African countries included in Haile (2018) [40] were: Egypt, Ghana, Kenya, Nigeria, and Rwanda.

**Table 3:** List of unique terms used to describe mobile applications related to female reproductive health.

Author(s) (Year)	Unique Term
Al-Rshoud (2021) [3]	Fertility tracking mobile applications
Andelsman (2021) [22]	2. App assisted period tracking
Anderson (2016) [23]	3. Mobile health apps
Berglund Scherwitzl (2016, 2017) [24,69]	4. Mobile based application
Blair (2021), [25] Gambier-Ross (2018) [37]	5. Fertility tracking application (FTA)
Blair (2021) [25]	6. Natural conception prediction app (NCPA)
Bradley (2021), [26] Jacobson (2018) [46]	7. Mobile app
Broad (2022) [15]	8. Period tracker apps
Costa Figueiredo (2021) [28]	9. Consumer facing fertility apps
Dietrich (2017) [30]	10. Device users, Smartphone apps for natural contraception
Epstein (2017) [6]	11. Personal informatics tools
Figueiredo (2018), [32] Homewood (2020) [45]	12. Self-tracking
Ford (2020) [33]	13. Female reproductive health apps
Ford (2022) [34]	14. Fertility-based reproductive health apps
Fox (2020) [35]	15. Mobile apps for menstrual cycle documentation, period tracking technology/mobile applications
French (2022) [36]	16. Fertility awareness technology
Gazibara (2020) [38]	17. Apps to track menstrual cycle
Hamper (2020) [41]	18. Fertility tracking smartphone applications, or fertility apps
Hohmann-Marriott (2021), (2022), (2023), [42,43,44] Ko (2023) [16]	19. Menstrual app
Karasneh (2020) [8]	20. Smartphone apps related to period tracking
Lee (2019) [49]	21. Menstrual health mobile apps
Levy (2018) [52]	22. App-supported menstrual tracking
Levy (2019) [7]	23. Period-tracking apps
MacKrill (2020) [53]	24. Menstrual-monitoring app
Ozcelik (2020) [55]	25. Smartphone application
Richman (2014) [57]	26. Mobile health application
Riley (2022) [58]	27. Menstrual tracking app (MTA)
Robertson (2021) [59]	28. Digital support tools for fertility
Schantz (2021) [11]	29. Menstrual cycle tracking app (MCTA)
Song (2018) [61]	30. Mobile application
Stanford (2020) [63]	31. Mobile computing apps
Starling (2018) [64]	32. Fertility app
Tuli (2022) [66]	33. Digital menstrual trackers
Zampino (2019) [68]	34. Self-tracking technologies

## Data Availability

The data presented in this study are available on request from the corresponding author.

## Supplementary Materials

The following supporting information can be downloaded at: https://www.mdpi.com/article/10.3390/healthcare13080877/s1, Table S1: Preferred Reporting Items for Systematic reviews and Meta-Analyses extension for Scoping Reviews (PRISMA–ScR) checklist.

## Appendix A

### Database Search Strategy

Database(s): Ovid MEDLINE(R) ALL 1946 to 24 April 2023

Search Strategy:

**#**	**Searches**	**Results**
1	Fertility/or Menstrual Cycle/	57,848
2	(menstru* or “period track*” or fertility).kf,tw.	158,304
3	1 or 2	177,575
4	Smartphone/ or Mobile Applications/	58,337
5	(tracker* or tracking or app or apps).kf,tw.	156,438
6	4 or 5	165,751
7	3 and 6	543
8	limit 7 to animals	84
9	7 not 8	459
10	limit 9 to yr = “2010–Current”	388

Database(s): Embase 1974 to 24 April 2023

Search Strategy:

**#**	**Searches**	**Results**
1	female fertility/or menstrual cycle/	53,990
2	(menstru* or “period track*” or fertility).kf,tw.	193,189
3	1 or 2	210,381
4	mobile phone/or smartphone/or mobile application/	60,818
5	(tracker* or tracking or app or apps).kf,tw.	216,193
6	4 or 5	259,941
7	3 and 6	1106
8	limit 7 to embase	538
9	limit 8 to yr = “2010–Current”	461
10	limit 9 to animals	36
11	9 not 10	425

**Scopus: searched 25 April 2023**
  (TITLE-ABS-KEY (menstru* OR “period track*” OR fertility) AND TITLE-ABS-KEY (tracker* OR tracking OR app OR apps)) AND PUBYEAR > 2009 AND PUBYEAR < 2024 AND (LIMIT-TO (DOCTYPE, “ar”) OR LIMIT-TO (DOCTYPE, “cp”)) AND (EXCLUDE (EXACTKEYWORD, “animals”))
  Results: 565
  **ACM Digital Library: searched 25 April 2023**
  AllField:(menstru* or “period track*” or fertility) AND All Field:(tracker* or tracking or app or apps); Applied filters: 2010–2023
  Results: 1367
  **IEEE Xplore: searched 26 April 2023**
  (“All Metadata”:menstru* OR “All Metadata”:”period tracker” OR “All Metadata”:”period trackers” OR “All Metadata”:”period tracking” OR “All Metadata”:fertility) AND (“All Metadata”:tracker* OR “All Metadata”:tracking OR “All Metadata”:app OR “All Metadata”:apps)
  Date limit: 2010–2023
  Results: 66
